# Supplementary material for: Metabolic Features of Saliva in Breast Cancer Patients
Source: Metabolites. 2022 Feb 10;12(2):166. doi: 10.3390/metabo12020166 (PMC8879753; doi:10.3390/metabo12020166)
Supplement: Supplementary file 1 [file metabolites-12-00166-s001.zip › metabolites-1575209-supplementary.pdf]

Table S1. Biochemical composition of saliva in breast cancer patients and healthy controls (full list).

Indicator	Breast Cancer, n=487	Control Group, n=298	p-value
pH	6.48 [6.24; 6.72]	6.49 [6.30; 6.72]	0.8763
Calcium, mmol/L	1.24 [0.87; 1.63]	1.27 [1.03; 1.55]	0.3566
Phosphorus, mmol/L	4.55 [3.47; 5.95]	4.36 [3.31; 5.85]	0.2804
Sodium, mmol/L	8.0 [4.9; 12.8]	8.4 [5.6; 12.1]	0.1875
Potassium, mmol/L	11.2 [8.0; 14.5]	10.8 [8.8; 14.2]	0.9744
Chlorides, mmol/L	25.7 [20.0; 32.3]	25.5 [20.7; 31.4]	0.4969
Magnesium, mmol/L	0.301 [0.235; 0.379]	0.296 [0.241; 0.356]	0.4505
Protein, mg/mL	0.64 [0.37; 1.09]	1.08 [0.65; 1.70]	0.0000
Urea, mmol/L	9.63 [6.25; 13.38]	6.66 [4.36; 9.13]	0.0000
Uric acid, µmol/L	65.4 [24.1; 136.1]	85.9 [34.4; 144.5]	0.0119
Albumin, mg/mL	0.305 [0.182; 0.551]	0.264 [0.175; 0.442]	0.0564
ALT, U/L	3.92 [2.77; 5.15]	3.85 [2.85; 5.08]	0.6724
AST, U/L	6.00 [4.17; 8.17]	5.58 [3.67; 7.50]	0.1117
AST/ALT-ratio, c.u.	1.48 [1.15; 1.92]	1.40 [1.13; 1.88]	0.2170
α-Aminoacids, mmol/L	4.23 [3.89; 4.76]	4.06 [3.83; 4.32]	0.0000
Imidazole compounds, mmol/L	0.281 [0.182; 0.402]	0.303 [0.205; 0.410]	0.0750
NO, µmol/L	29.1 [17.4; 44.6]	22.8 [13.2; 36.8]	0.0001
ALP, U/L	72.8 [47.8; 106.5]	60.8 [41.3; 84.7]	0.0002
MM 254, c.u.	0.247 [0.164; 0.380]	0.274 [0.179; 0.383]	0.1521
MM 280, c.u.	0.198 [0.140; 0.326]	0.224 [0.147; 0.324]	0.2038
LDH, U/L	1451.0 [861.6; 2093.0]	1101.5 [635.7; 1908.0]	0.0002
Catalase, nkat/mL	3.78 [2.53; 5.99]	4.58 [3.32; 5.79]	0.0052
Sialic acids, mmol/L	0.201 [0.140; 0.275]	0.183 [0.128; 0.293]	0.5455
Pyruvic acid, µmol/L	14.46 [10.05; 19.61]	12.99 [9.56; 18.14]	0.0551
Diene conjugates, c.u.	3.93 [3.72; 4.13]	3.92 [3.78; 4.07]	0.9332
Triene conjugates, c.u.	0.897 [0.789; 1.020]	0.893 [0.818; 0.994]	0.9102
Schiff bases, c.u.	0.541 [0.484; 0.673]	0.545 [0.510; 0.576]	0.7607
MDA, µmol/L	7.09 [5.81; 8.97]	6.50 [5.73; 7.95]	0.0006
GGT, U/L	23.2 [20.0; 26.5]	20.4 [17.4; 24.4]	0.0000
Seromucoids, c.u.	0.097 [0.062; 0.155]	0.091 [0.061; 0.130]	0.0793
Superoxide dismutase, c.u.	73.7 [34.2; 142.1]	57.9 [31.6; 113.2]	0.0247
α-Amylase, U/L	306.5 [122.6; 605.3]	185.2 [83.5; 384.4]	0.0002
Antioxidant activity, mmol/L	2.32 [1.43; 3.35]	2.32 [1.41; 3.62]	0.3426
Lactic acid, mmol/L	1.67 [1.41; 2.09]	1.78 [1.48; 2.28]	0.1899
Peroxidase, c.u.	0.440 [0.250; 0.880]	0.400 [0.170; 0.750]	0.3085
Na/K-ratio, c.u.	0.686 [0.481; 1.067]	0.751 [0.505; 1.190]	0.1270
Ca/P-ratio, c.u.	0.268 [0.192; 0.371]	0.278 [0.214; 0.381]	0.0748
SOD/Catalase-ratio, c.u.	19.8 [8.4; 39.7]	14.5 [6.6; 37.7]	0.0168
SB/(DC+TC)-ratio, c.u.	0.111 [0.101; 0.134]	0.112 [0.108; 0.118]	0.5248
SB/TC-ratio, c.u.	0.627 [0.561; 0.704]	0.609 [0.567; 0.667]	0.0503

Table S2. Comparison of saliva composition depending on breast cancer stage and healthy control (Kruskal-Wallis criterion) .

Indicator	Group I, n=226	Group II, n=131	Group III, n=75	Group IV, n=55	Kruskal-Wallis test (H, p)
pH	6.50 [6.24; 6.75]	6.45 [6.28; 6.63]	6.43 [6.17; 6.73]	6.61 [6.27; 6.87]	7.028; 0.1344
Calcium, mmol/L	1.23 [0.84; 1.63]	1.25 [0.90; 1.69]	1.21 [1.02; 1.57]	1.26 [0.69; 1.52]	1.131; 0.8893
Phosphorus, mmol/L	4.64 [3.54; 5.99]	4.44 [3.42; 5.87]	4.39 [2.63; 5.44]	4.79 [3.62; 6.28]	6.583; 0.1597
Sodium, mmol/L	8.0 [4.9; 12.9]	8.2 [4.5; 13.2]	7.1 [5.2; 11.1]	8.2 [6.1; 12.0]	2.175; 0.7035
Potassium, mmol/L	11.8 [8.9; 14.2]	10.3 [7.2; 14.6]	10.7 [7.3; 14.1]	12.0 [9.0; 14.7]	3.747; 0.4413
Chlorides, mmol/L	25.5 [20.0; 31.6]	25.7 [20.3; 31.6]	28.0 [20.4; 32.8]	25.9 [18.8; 35.6]	1.481; 0.8301
Magnesium, mmol/L	0.282 [0.219; 0.380]	0.296 [0.230; 0.369]	0.318 [0.242; 0.377]	0.345 [0.254; 0.395]	6.849; 0.1441
Protein, mg/mL	0.68 [0.39; 1.08]	0.56 [0.37; 1.07]	0.58 [0.29; 1.14]	0.84 [0.46; 1.16]	71.46; 0.0000
Urea, mmol/L	10.08 [6.66; 14.12]	9.18 [5.90; 13.10]	9.17 [6.00; 12.08]	8.37 [5.83; 12.30]	71.86; 0.0000
Uric acid, µmol/L	59.6 [23.2; 128.4]	58.3 [26.3; 126.1]	75.6 [16.7; 137.6]	103.8 [39.5; 141.3]	10.87; 0.0280
Albumin, mg/mL	0.315 [0.179; 0.608]	0.296 [0.184; 0.498]	0.274 [0.149; 0.457]	0.335 [0.204; 0.507]	7.265; 0.1225
ALT, U/L	4.00 [2.77; 5.23]	4.00 [2.69; 5.23]	3.92 [2.85; 4.92]	3.54 [2.46; 4.88]	2.523; 0.6406
AST, U/L	6.00 [4.08; 8.25]	6.13 [4.33; 8.50]	5.71 [4.08; 7.75]	5.71 [3.92; 7.75]	4.314; 0.3651
AST/ALT-ratio, c.u.	1.44 [1.13; 1.94]	1.47 [1.15; 1.90]	1.52 [1.18; 1.92]	1.54 [1.16; 1.97]	1.904; 0.7534
α-Aminoacids, mmol/L	4.29 [3.90; 4.89]	4.23 [3.91; 4.67]	4.14 [3.86; 4.72]	4.24 [3.84; 4.65]	25.64; 0.0000
Imidazole compounds, mmol/L	0.281 [0.182; 0.395]	0.288 [0.190; 0.417]	0.303 [0.182; 0.417]	0.258 [0.167; 0.387]	5.193; 0.2681
NO, µmol/L	29.1 [17.2; 47.5]	30.4 [18.2; 42.5]	22.8 [13.9; 46.0]	32.3 [22.1; 44.6]	18.41; 0.0010
ALP, U/L	76.1 [50.0; 108.7]	68.4 [47.8; 104.3]	71.7 [41.3; 102.1]	82.6 [47.8; 123.9]	16.35; 0.0026
MM 254, c.u.	0.257 [0.168; 0.389]	0.245 [0.151; 0.382]	0.191 [0.144; 0.356]	0.247 [0.160; 0.383]	5.543; 0.2360
MM 280, c.u.	0.216 [0.146; 0.331]	0.205 [0.126; 0.329]	0.175 [0.126; 0.289]	0.201 [0.142; 0.333]	5.294; 0.2585
LDH, U/L	1509.0	1452.5	1280.0	1703.0	16.79; 0.0021

	[829.5; 2088.0]	[899.1; 2070.0]	[636.8; 2034.0]	[972.0; 2279.0]	
Catalase, nkat/mL	3.86 [2.65; 6.25]	3.70 [2.36; 5.22]	3.84 [2.25; 6.11]	3.52 [2.56; 5.51]	404.7; 0.0000
Sialic acids, mmol/L	0.189 [0.140; 0.262]	0.217 [0.146; 0.299]	0.207 [0.128; 0.299]	0.183 [0.110; 0.250]	4.888; 0.2990
Pyruvic acid, µmol/L	14.95 [10.29; 20.10]	14.22 [10.29; 19.12]	12.99 [7.84; 18.38]	13.60 [10.66; 19.36]	6.106; 0.1913
Diene conjugates, c.u.	3.94 [3.73; 4.18]	3.89 [3.70; 4.10]	3.89 [3.69; 4.06]	3.97 [3.79; 4.16]	3.659; 0.4541
Triene conjugates, c.u.	0.896 [0.795; 1.017]	0.864 [0.785; 0.995]	0.899 [0.794; 1.029]	0.913 [0.786; 1.054]	2.475; 0.6491
Schiff bases, c.u.	0.546 [0.479; 0.671]	0.529 [0.483; 0.667]	0.536 [0.477; 0.625]	0.565 [0.502; 0.769]	4.587; 0.3323
MDA, µmol/L	6.92 [5.73; 8.89]	7.09 [5.90; 9.15]	7.18 [5.94; 9.23]	7.26 [5.81; 8.89]	13.56; 0.0089
GGT, U/L	23.1 [19.8; 26.1]	24.0 [20.4; 27.4]	22.7 [19.9; 26.6]	22.2 [19.4; 26.1]	37.86; 0.0000
Seromucoids, c.u.	0.099 [0.062; 0.163]	0.104 [0.068; 0.165]	0.080 [0.055; 0.135]	0.098 [0.055; 0.138]	8.257; 0.0826
Superoxide dismutase, c.u.	76.3 [34.2; 168.4]	57.9 [28.9; 113.2]	75.0 [36.8; 134.2]	73.7 [39.5; 113.2]	10.31; 0.0355
α-Amylase, U/L	319.0 [157.7; 596.8]	262.2 [124.8; 664.9]	341.5 [111.6; 940.6]	347.8 [115.4; 482.9]	14.84; 0.0050
Antioxidant activity, mmol/L	2.31 [1.50; 3.38]	2.42 [1.59; 3.44]	2.35 [1.43; 3.09]	1.92 [1.21; 3.63]	1.899; 0.7542
Lactic acid, mmol/L	1.67 [1.42; 2.10]	1.60 [1.34; 2.10]	1.69 [1.42; 1.98]	1.68 [1.49; 2.10]	1.865; 0.7606
Peroxidase, c.u.	0.455 [0.250; 0.925]	0.440 [0.250; 0.750]	0.380 [0.170; 0.760]	0.560 [0.300; 0.790]	3.024; 0.5539
Na/K-ratio, c.u.	0.698 [0.461; 1.036]	0.649 [0.494; 1.016]	0.700 [0.487; 1.217]	0.747 [0.473; 1.090]	2.653; 0.6174
Ca/P-ratio, c.u.	0.256 [0.185; 0.354]	0.274 [0.196; 0.380]	0.274 [0.221; 0.450]	0.266 [0.185; 0.345]	10.40; 0.0343
SOD/Catalase-ratio, c.u.	22.1 [8.2; 41.7]	17.9 [6.7; 31.0]	18.0 [9.1; 50.0]	19.5 [10.2; 39.5]	7.890; 0.0957
SB/(DC+TC)-ratio, c.u.	0.111 [0.100; 0.133]	0.110 [0.101; 0.131]	0.111 [0.102; 0.125]	0.114 [0.107; 0.151]	4.182; 0.3819
SB/TC-ratio, c.u.	0.628 [0.557; 0.698]	0.627 [0.566; 0.696]	0.601 [0.556; 0.683]	0.647 [0.569; 0.765]	**9.531; 0.0491**

Table S3. Comparison of the saliva composition of patients with ductal and lobular breast cancer and healthy controls (Kruskal-Wallis criterion) .

Indicator	Lobular BC, n=86	Ductal BC, n=227	Kruskal-Wallis test (H, p)
pH	6.51 [6.19; 6.76]	6.50 [6.29; 6.76]	0.7412; 0.6903
Calcium, mmol/L	1.20 [0.93; 1.64]	1.25 [0.83; 1.59]	0.8670; 0.6482
Phosphorus, mmol/L	4.58 [3.47; 5.74]	4.51 [3.46; 6.03]	0.9079; 0.6351
Sodium, mmol/L	6.5 [4.1; 11.8]	7.7 [4.7; 12.5]	5.183; 0.0749
Potassium, mmol/L	9.9 [6.5; 13.1]	10.9 [8.0; 14.2]	3.736; 0.1545
Chlorides, mmol/L	25.6 [18.8; 32.2]	25.1 [19.2; 32.6]	0.2796; 0.8695
Magnesium, mmol/L	0.295 [0.236; 0.371]	0.292 [0.235; 0.366]	0.4977; 0.7797
Protein, mg/mL	0.63 [0.36; 1.02]	0.69 [0.40; 1.15]	49.97; 0.0000
Urea, mmol/L	9.86 [4.86; 14.33]	9.43 [6.03; 12.83]	41.49; 0.0000
Uric acid, µmol/L	56.73 [27.03; 126.92]	70.27 [25.00; 140.48]	4.922; 0.0853
Albumin, mg/mL	0.296 [0.187; 0.419]	0.313 [0.177; 0.590]	3.865; 0.1448
ALT, U/L	3.65 [2.50; 5.12]	3.77 [2.69; 5.15]	0.6820; 0.7111
AST, U/L	4.83 [3.27; 7.67]	5.96 [4.25; 8.08]	3.881; 0.1432
AST/ALT-ratio, c.u.	1.37 [1.13; 1.90]	1.53 [1.15; 1.94]	1.944; 0.3782
α-Aminoacids, mmol/L	4.16 [3.89; 4.79]	4.29 [3.88; 4.89]	20.75; 0.0000
Imidazole compounds, mmol/L	0.266 [0.190; 0.379]	0.273 [0.175; 0.395]	4.668; 0.0969
NO, µmol/L	25.4 [15.6; 44.6]	26.8 [18.2; 40.7]	8.614; 0.0135
ALP, U/L	69.54 [39.11; 97.79]	73.88 [47.81; 108.65]	12.47; 0.0020
MM 254, c.u.	0.206 [0.136; 0.332]	0.256 [0.168; 0.382]	8.728; 0.0127
MM 280, c.u.	0.177 [0.118; 0.291]	0.200 [0.144; 0.342]	7.210; 0.0272
LDH, U/L	1374.0 [731.8; 2008.0]	1532.0 [1022.0; 2217.0]	20.12; 0.0000
Catalase, nkat/mL	3.26 [2.45; 5.49]	3.88 [2.52; 6.25]	7.107; 0.0286
Sialic acids, mmol/L	0.192 [0.140; 0.275]	0.189 [0.134; 0.269]	0.0608; 0.9701
Pyruvic acid, µmol/L	12.62 [8.58; 18.63]	14.46 [10.05; 20.10]	4.965; 0.0835
Diene conjugates, c.u.	3.91 [3.66; 4.08]	3.93 [3.74; 4.18]	1.876; 0.3914
Triene conjugates, c.u.	0.930 [0.822; 1.103]	0.904 [0.800; 1.031]	3.821; 0.1480
Schiff bases, c.u.	0.576 [0.490; 0.755]	0.555 [0.494; 0.686]	6.924; 0.0314
MDA, µmol/L	6.92 [5.47; 8.29]	7.14 [5.90; 9.15]	10.13; 0.0063
GGT, U/L	21.9 [18.3; 24.9]	23.4 [19.8; 26.5]	27.94; 0.0000
Seromucoids, c.u.	0.093 [0.055; 0.147]	0.099 [0.063; 0.162]	4.321; 0.1153
Superoxide dismutase, c.u.	84.2 [31.6; 152.6]	68.4 [39.5; 144.7]	6.430; 0.0402
α-Amylase, U/L	217.4 [113.4; 451.5]	304.7 [116.0; 526.4]	7.360; 0.0252
Antioxidant activity, mmol/L	2.46 [1.29; 3.52]	2.23 [1.19; 3.18]	3.603; 0.1651
Lactic acid, mmol/L	1.69 [1.34; 1.96]	1.68 [1.41; 2.14]	1.768; 0.4130
Peroxidase, c.u.	0.320 [0.210; 0.750]	0.555 [0.290; 0.865]	4.026; 0.1336

Na/K-ratio, c.u.	0.65 [0.46; 1.14]	0.67 [0.49; 1.03]	2.842; 0.2414
Ca/P-ratio, c.u.	0.27 [0.20; 0.36]	0.27 [0.19; 0.37]	2.023; 0.3636
SOD/Catalase-ratio, c.u.	21.5 [9.9; 41.7]	19.9 [8.2; 42.8]	6.739; 0.0344
SB/(DC+TC)-ratio, c.u.	0.115 [0.107; 0.153]	0.112 [0.103; 0.137]	4.345; 0.1139
SB/TC-ratio, c.u.	0.644 [0.579; 0.707]	0.631 [0.569; 0.723]	10.32; 0.0057
